# Loneliness during Covid-19: Does Living Situation or Ability to Access Information about Social Activities Matter?

**DOI:** 10.1093/geroni/igaa057.3481

**Published:** 2020-12-16

**Authors:** Patti Parker, Verena Menec, Nancy Newall

**Affiliations:** 1 University of Alberta, Edmonton, Alberta, Canada; 2 University of Manitoba, Winnipeg, Manitoba, Canada; 3 Brandon University, Brandon, Manitoba, Canada

## Abstract

Social isolation is deleterious for both mental and physical health (Coyle & Dugan, 2012; Hawkley et al., 2006). Conversely, social participation has mental and physical health benefits (Novek et al., 2013). In light of the current Covid-19 pandemic requiring social distancing, the present study examined whether living situation and ability to access information about social activities are associated with older adults’ loneliness during the pandemic. Specifically, we surveyed ninety-one adults aged 60 years or older in May and June of 2020, at a time when social distancing measures were still in place. We tested whether their living situation and having access to information about social activities was associated with loneliness. OLS regression analyses revealed living alone was associated with higher loneliness (b = .43, p = .050); and having access to information about social activities was associated with lower loneliness (b = -.18, p = .027) amidst the pandemic. The analyses controlled for participants’ age, gender, and education. Our findings highlight that during Covid-19, older adults’ living situation and access to information about social activities matter and may impact their social behavior. Thus, at this difficult time, it is recommended organizations that offer social activities find creative ways to reach those living alone who will benefit most from having access to such activities.

